# In vivo TurboID labeling for paired proteomic and transcriptomic profiling of neurons and astrocytes

**DOI:** 10.1002/alz.091147

**Published:** 2025-01-03

**Authors:** Christina C Ramelow, Hailian Xiao, Lihong Cheng, Prateek Kumar, Eric B. Dammer, Maureen Sampson, Ruth S Nelson, Sneha Malepati, Qi Quo, Pritha Bagchi, Duc M Duong, Nicholas T Seyfried, Steven A Sloan, Srikant Rangaraju

**Affiliations:** ^1^ Emory University School of Medicine, Atlanta, GA USA; ^2^ Yale University School of Medicine, New Haven, CT USA; ^3^ Emory University Center for Neurodegenerative Disease, Atlanta, GA USA

## Abstract

**Background:**

Our group has developed the innovative proximity labeling cell‐type specific in vivo biotinylation of proteins (CIBOP) approach to quantify cell‐specific in vivo proteomic and transcriptomic signatures that may lead to identify novel therapeutic targets for Alzheimer’s disease (AD) pathogenesis. CIBOP uses TurboID, a biotin ligase, selectively expressed in the cell type of interest using a conditional Cre/lox genetic strategy to label the cytosolic proteome. Using mass spectrometry (MS)‐based proteomics, we have found that TurboID biotinylates many RNA‐binding and ribosomal proteins. We extended the CIBOP approach to obtain representative cell type‐specific transcriptomes and proteomes.

**Method:**

We crossed cell‐specific Cre lines (astrocytic: Aldh1l1‐Cre‐ert2 and neuronal: Camk2a‐Cre‐ert2) and Rosa26^TurboID/wt^ floxed mice for cell‐specific proteomic labeling (astrocyte‐CIBOP and neuron‐CIBOP). CIBOP and control (Cre‐only) mice received tamoxifen, followed by biotin‐containing water. While maintaining RNA‐protein interactions, cortical tissue was lysed, biotinylated proteins were enriched via streptavidin beads, and RNA and proteins were eluted. Immunofluorescent microscopy (IF), biochemical assays, MS‐based proteomics, and RNA‐sequencing were completed to confirm cell‐specific molecular profiling. Concordance analysis of paired proteomes and transcriptomes from CIBOP mouse brains was conducted.

**Result:**

Western blot analysis of the cortex confirmed biotinylation of the cellular proteome of CIBOP mice when compared to controls. Cell‐type specificity was further validated by IF images showing that biotin‐labeled proteins colocalized with corresponding astrocytic (e.g., GFAP and NDRG2) or neuronal markers (e.g., MAP2 and beta‐tubulin‐3). RNA gel electrophoresis displayed high levels of RNA from CIBOP brain streptavidin pulldowns and low RNA yield from control pulldowns. MS‐based proteomics and RNA‐sequencing analysis showed upregulation of astrocytic proteins (e.g., Hepacam, Glu, Aqp4, Plpp3) and genes (e.g., *Sox9*, *Aqp4*, *Gfap, Apoe*) from astrocyte‐CIBOP brain. In contrast, upregulation of neuronal proteins (e.g., Map2, Ncam1, Mapt) and genes (e.g., *Pdyn*, *Tmem130*, *Ptpn7*) was observed in neuron‐CIBOP brain samples, confirming specificity.

**Conclusion:**

Together, these results validate the CIBOP approach to capturing the cortical Aldh1l1‐positive astrocytic and Camk2a‐positive neuronal proteome and transcriptome. Our innovative in vivo cell type‐specific and native‐state dual‐omics approach provides complementary transcriptomic and proteomic information that can extend to AD models to investigate disease mechanisms, discover new biomarkers, and identify therapeutic targets.

